# Trends in Donation After Circulatory Death in Lung Transplantation in the United States: Impact Of Era

**DOI:** 10.3389/ti.2022.10172

**Published:** 2022-04-04

**Authors:** Christopher M. Bobba, Bryan A. Whitson, Matthew C. Henn, Nahush A. Mokadam, Brian C. Keller, Justin Rosenheck, Asvin M. Ganapathi

**Affiliations:** ^1^ Division of Thoracic and Cardiovascular Surgery, University of Florida Health, Gainesville, FL, United States; ^2^ Division of Cardiac Surgery, Department of Surgery, The Ohio State University Wexner Medical Center, Columbus, OH, United States; ^3^ Division of Pulmonary, Critical Care and Sleep Medicine, Department of Internal Medicine, The Ohio State University Wexner Medical Center, Columbus, OH, United States

**Keywords:** lung transplantation, organ procurment, organ donation, donation after cardiac death, donation after brain death

## Abstract

**Background:** Use of lungs donated after circulatory death (DCD) has expanded, but changes in donor/recipient characteristics and comparison to brain dead donors (DBD) has not been studied. We examined the evolution of the use of DCD lungs for transplantation and compare outcomes to DBD lungs.

**Methods:** The SRTR database was used to construct three 5-year intervals. Perioperative variables and survival were compared by era and for DCD vs. DBD. Geographic variation was estimated using recipient permanent address.

**Results:** 728 DCD and 27,205 DBD lung transplants were identified. DCD volume increased from Era 1 (*n* = 73) to Era 3 (*n* = 528), representing 1.1% and 4.2% of lung transplants. Proportionally more DCD recipients were in ICU or on ECMO pre-transplant, and had shorter waitlist times. DCD donors were older, had lower PaO2/FiO2 ratios compared to DBD, more likely to be bilateral, had longer ischemic time, length of stay, post-op dialysis, and increased use of lung perfusion. There was no difference in overall survival. Geographically, use was heterogeneous.

**Conclusion:** DCD utilization is low but increasing. Despite increasing ischemic time and transplantation into sicker patients, survival is similar, which supports further DCD use in lung transplantation. DCD lung transplantation presents an opportunity to continue to expand the donor pool.

## Introduction

Lung transplantation remains the gold standard therapy for end stage lung disease, however a shortage of viable organs remains (1, 2). Use of lungs from donors after circulatory death (DCD) has been instrumental in increasing organ supply. DCD use has increased to 4.8% of all lung transplants in 2018, and expansion in their use is an avenue for continued growth in available organs (3). Evidence demonstrating equivalent outcomes to donation after brain death (DBD) organs has led to increased utilization with similar mortality, primary graft dysfunction (PGD) and acute rejection rates at 1 and 5 years (4–7). These results are encouraging, however, studies have not performed an analysis of the DCD cohort compared to DBD over time including a profile of DCD lung donors and recipients in the United States.

Given the importance of DCD lungs in expanding the donor pool and some evidence of their equivalence to traditional DBD organs, a more thorough analysis is warranted. We hypothesized that geographic and individual center variation in the usage of DCD lungs still exists and that differences exist between DBD and DCD donors. Further, we anticipate that the profile of the recipients of DCD organs has changed over time and represents a more heterogeneous cohort than early experiences with DCD lung transplantation. In this study, we sought to characterize the evolving nature of the use of DCD organs for lung transplant, and compare donor, recipient, and operative characteristics with traditional DBD organs.

## Methods

### Data Source and Patient Population

This retrospective cohort study utilized data from the United Network for Organ Sharing/Organ Procurement and Transplant Network (UNOS/OPTN) STAR file. The UNOS/OPTN STAR file is a well validated dataset of patients undergoing transplantation in the United States (1, 8). The study was submitted for Ohio State IRB approval (protocol: 2018H0079) and deemed exempt. The STAR file was queried from 5/1/2005 to 4/30/2020 to include all DCD and DBD lung transplants after implementation of the lung allocation score (LAS) in 2005. DCD and DBD recipient outcomes were collected using the identifier of NON_HRT_DON in the STAR file. Pediatric patients (age <18), those with a previous lung transplant, and multi-organ transplants were excluded from analysis. Three “eras” were constructed based on date of transplantation in 5-year increments: 5/1/2005-4/30/2010 (**era 1**), 5/1/2010-4/30/2015 (**era 2**), and 5/1/2015-4/30/2020 (**era 3**). We included all instances of DCD lung donation, including controlled and uncontrolled DCD. For purposes of geographic variations, the state of recipient permanent address was utilized to identify where usage of a DCD organ occurred.

### Statistical Analysis

Data was analyzed for normality using the Kolmogorov-Smirnov test and missingness for each variable was calculated (Supplementary Table S1). Continuous data was compared with an analysis of variance (ANOVA) or the Kruskal-Wallis test for parametric and non-parametric data respectively. Categorical variables were compared using the Chi-Square test. Survival rates were calculated simultaneously across all 3 eras using the Kaplan-Meier method and the log-rank test. An additional Kaplan-Meier survival analysis examined each era of recipients of DCD lungs as compared to recipients of DBD lungs. A Cox proportional hazard model was created to examine the independent effect of era and DCD donors on survival. This model utilized the following covariates which were selected a priori: DCD status, era, LAS, age, body mass index (BMI), sex, waitlist time, diabetes, smoking history, pre-operative hospitalization status, yearly center volume, organ ischemic time, organ distance traveled, donor BMI, and donor age. De-identified recipient center ID numbers were used to determine center DCD lung transplantation volume. DCD utilization by state was determined according to recipient permanent address.

Missing data was excluded from analysis and no imputation was performed. In all cases *p* < 0.05 was considered significant. All statistical analysis was performed using R version 3.6.2 (Vienna, Austria).

## Results

### Recipient, Donor and Operative Characteristics of DCD and DBD Organs

A total of 27,205 DBD organ, and 728 DCD organ lung transplants were identified from 5/1/2005 to 4/30/2020. Recipients of DCD organs were slightly older (61 vs. 60 years old, *p* < 0.01), more likely to be in the ICU prior to transplant (13.9% vs. 10.7%, *p* < 0.01) and more likely to require pre-operative ECMO (6.9% vs. 3.7%, *p* < 0.01) (Table 1). DCD organ donors were older (39 vs. 33 years, *p* < 0.01), more commonly Caucasian (81.7% vs. 61.2%, *p* < 0.01), had higher BMI (26.3 vs. 25.3, *p* < 0.01), and lower mean PaO2:FiO2 (PF) ratio (423 vs. 436, *p* < 0.01) than their DBD counterparts. In the DCD cohort, the inciting event leading to becoming a donor was more likely to be anoxia (39.3% vs. 21.6%, *p* < 0.01) and less likely trauma (31.2% vs. 43.6%, *p* < 0.01) than in the DBD organ cohort (Table 2).

**TABLE 1:** Recipient demographics and baseline characteristics.

Variable	Overall	DBD	DCD	P-value
Cohort Size	*27,933*	*27,205*	*728*	
Age (y)	60 (51, 65)	60 (51, 65)	61 (53, 66)	0.01*
Male Sex	16,645 (59.6%)	16,194 (59.5%)	451 (62%)	0.20
Ethnicity				0.02*
Caucasian	22,779 (81.5%)	22,163 (81.5%)	616 (84.6%)	
African-American	2,524 (9%)	2,459 (9%)	65 (8.9%)	
Other	2,630 (9.4%)	2,583 (9.5%)	47 (6.5%)	
BMI (kg/m2)	25.7 (22, 29)	25.7 (22, 29)	25.4 (22, 28.7)	0.28
Former Smoker	16,551 (60.3%)	16,119 (60.3%)	432 (59.8%)	0.81
Diabetes	5,229 (18.8%)	5,088 (18.8%)	141 (19.4%)	0.74
GFR (mL/min/1.73m2)	92.8 (73.4, 120.4)	92.8 (73.4, 120.3)	96.1 (72.8, 122.1)	0.33
Diagnosis				0.70
Cystic Fibrosis/Immunodeficiency	3,028 (10.8%)	2,952 (10.9%)	76 (10.4%)	
Obstructive Lung disease	8,360 (29.9%)	8,128 (29.9%)	232 (31.9%)	
Pulmonary Vascular disease	1,076 (3.9%)	1,050 (3.9%)	26 (3.6%)	
Restrictive Lung disease	15,469 (55.4%)	15,075 (55.4%)	394 (54.1%)	
Blood Group				0.11
A	11,164 (40%)	10,861 (39.9%)	303 (41.6%)	
B	3,107 (11.1%)	3,039 (11.2%)	68 (9.3%)	
AB	1,082 (3.9%)	1,063 (3.9%)	19 (2.6%)	
O	12,580 (45%)	12,242 (45%)	338 (46.4%)	
Medical Condition				0.03*
Not Hospitalized	22,140 (80.1%)	21,578 (80.2%)	562 (77.2%)	
Hospitalized	2,508 (9.1%)	2,443 (9.1%)	65 (8.9%)	
In ICU	2,985 (10.8%)	2,884 (10.7%)	101 (13.9%)	
Functional Status				0.08
ADL With No Assistance	6,140 (22.5%)	5,986 (22.6%)	154 (21.5%)	
ADL With Assistance	10,379 (38.1%)	10,078 (38%)	301 (42%)	
Disabled/Hospitalized	10,741 (39.4%)	10,480 (39.5%)	261 (36.5%)	
On Ventilator	1,567 (5.6%)	1,523 (5.6%)	44 (6%)	0.66
LAS	40.2 (34.8, 51.8)	40.2 (34.8, 51.8)	39.1 (34.2, 51.7)	0.10
PRA	0 (0, 1)	0 (0, 1)	0 (0, 0)	0.02*
Days on Waitlist	59 (17, 184)	60 (17, 184)	49 (14, 175)	0.05*
Previous ECMO/on ECMO	1,051 (3.8%)	1,001 (3.7%)	50 (6.9%)	0.01*

Data displayed as mean ± standard deviation (SD) median (interquartile range) for parametric or non-parametric continuous variables respectively and number (percent of total) for categorical variables. BMI, body mass index; DBD, donation after brain death; DCD, donation after circulatory death; GFR, glomerular filtration rate; ICU, intensive care unit; ADL, activities of daily living; LAS, lung allocation score; PRA, percent reactive antibodies; ECMO, extracorporeal membrane oxygenation. * indicates *p* < 0.05.

**TABLE 2:** Donor characteristics.

Variable	Overall	DBD	DCD	P-Value
Cohort Size	27,933	27,205	728	
Age	33 (23, 46)	33 (22, 46)	39 (28, 48)	< 0.01*
Male Sex	16,888 (60.5%)	16,455 (60.5%)	433 (59.5%)	0.61
Ethnicity				< 0.01*
Caucasian	17,243 (61.7%)	16,648 (61.2%)	595 (81.7%)	
African-American	5,177 (18.5%)	5,128 (18.8%)	49 (6.7%)	
Other	5,513 (19.8%)	5,429 (20.0%)	84 (11.6%)	
BMI (kg/m^2^)	25.3 (22.4, 28.9)	25.3 (22.4, 28.9)	26.3 (23, 31)	< 0.01*
Coronary artery disease	1,487 (5.4%)	1,479 (5.5%)	8 (1.1%)	< 0.01*
Smoking History	2,595 (9.4%)	2,542 (9.5%)	53 (7.4%)	0.07
Recent cocaine Use	4,168 (15.2%)	4,022 (15.1%)	146 (20.2%)	0.01*
Diabetes	2,041 (7.3%)	1,990 (7.4%)	51 (7%)	0.80
Hypertension	6,540 (23.6%)	6,355 (23.5%)	185 (25.6%)	0.21
Inciting Event Leading to Donation				< 0.01*
Anoxia	6,154 (22%)	5,868 (21.6%)	286 (39.3%)	
CVA	8,856 (31.7%)	8,657 (31.8%)	199 (27.3%)	
Head Trauma	12,099 (43.3%)	11,872 (43.6%)	227 (31.2%)	
CNS Tumor	174 (0.6%)	173 (0.6%)	1 (0.1%)	
Other	649 (2.3%)	634 (2.3%)	15 (2.1%)	
Donor Bloodstream Infection	2,076 (7.4%)	2,019 (7.4%)	57 (7.8%)	0.73
Donor Clinical Infection	18,278 (66.4%)	17,785 (66.3%)	493 (68.1%)	0.34
Donor Pulmonary Infection	16,214 (58%)	15,778 (58%)	436 (59.9%)	0.33
PaO2/FiO2 Ratio	435.9 (373, 492)	436 (374, 492)	423 (360, 481)	< 0.01*

Data displayed as mean ± standard deviation (SD) median (interquartile range) for parametric or non-parametric continuous variables respectively and number (percent of total) for categorical variables. BMI, body mass index; CVA, cerebrovascular accident; CNS, central nervous system. * indicates *p* < 0.05.

Transplants utilizing DCD organs were more commonly bilateral lung transplants (76.9% vs. 69.8%, *p* < 0.01), had longer total ischemic time (6.3 vs. 5.1 h, *p* < 0.01), longer post-operative length of stay (21 vs. 16 days, *p* < 0.01), more commonly required dialysis (11.1% vs. 6.5%, *p* < 0.01), and were more likely to use Ex-vivo lung perfusion (EVLP) (27.2% vs. 3.7%, *p* < 0.01). DCD organ transplants also more commonly occurred at centers with higher annual total lung transplant volume (55.6 vs. 39.2 average yearly center volume for centers utilizing DCD lungs vs. centers only performing DBD lung transplantation, *p* < 0.01) (Table 3).

**TABLE 3:** Operative characteristics and postoperative outcomes.

Variable	Overall	DBD	DCD	P-Value
Cohort Size	*27933*	*27205*	*728*	
Type of Transplant				< 0.01
Bilateral	19,544 (70%)	18,984 (69.8%)	560 (76.9%)	
Single	8,389 (30%)	8,221 (30.2%)	168 (23.1%)	
Distance Traveled (nautical miles)	140 (26, 313)	142 (26, 313)	113.5 (16, 325.2)	0.284
Ischemia Time (hours)	5.1 (4.1, 6.2)	5.1 (4.1, 6.2)	6.3 (5.1, 8.2)	< 0.01
Length of Stay (days)	16 (11, 27)	16 (11, 27)	21 (14, 37)	< 0.01
Postop Dialysis	1,821 (6.6%)	1,740 (6.5%)	81 (11.1%)	<0.01
Postop Stroke	618 (2.3%)	603 (2.3%)	15 (2.1%)	0.83
Postop Airway Dehiscence	414 (1.5%)	398 (1.5%)	16 (2.2%)	0.16
In-Hospital Mortality	1,255 (4.6%)	1,213 (4.6%)	42 (5.9%)	0.12
Acute Rejection Before Discharge				0.01
Yes & Treated with Immunosuppressant	1,999 (7.3%)	1,926 (7.2%)	73 (10%)	
Yes & Not Treated with Immunosuppressant	303 (1.1%)	293 (1.1%)	10 (1.4%)	
No	25,270 (91.7%)	24,625 (91.7%)	645 (88.6%)	
Rejection Treatment Within One Year	5,657 (26.5%)	5,514 (26.4%)	143 (28.6%)	0.30
Lung Perfusion Used	330 (4.8%)	241 (3.7%)	89 (27.2%)	<0.01

Data displayed as mean ± standard deviation (SD) median (interquartile range) for parametric or no-parametric continuous variables respectively and number (percent of total) for categorical variables. Lung perfusion data available from 2/28/2018-4/30/2020. * indicates *p* < 0.05.

### Recipient, Donor and Operative Characteristics of DCD Organs by Era

A total of 728 transplants using DCD lungs were identified across 3 eras with 73 transplants in era 1, 127 in era 2, and 528 in era 3. 2 donors (0.3%) were identified as uncontrolled DCD, and the remaining were controlled DCD. Median recipient age increased from 56 years (era 1) to 62 years (era 3) (*p* < 0.01) and there was an increase in disabled/hospitalized pre-operative functional status after the first era (era 1–15.5%, era 2–36.0%, era 3–39.4%; *p* < 0.01). Eras 2 and 3 had increased LAS (*p* < 0.01) and reduced waitlist time (*p* < 0.01). Additionally, later eras were associated with increases in transplant for restrictive lung disease (*p* < 0.01) (Table 4).

**TABLE 4:** Selected DCD characteristics by era.

Variable	Overall	Era 1	Era 2	Era 3	P-value
Date Range	*5/1/05 to 4/30/20*	*5/1/05 to 4/30/10*	*5/1/10 to 4/30/15*	*5/1/15 to 4/30/20*	
Cohort Size	*728*	*73*	*127*	*528*	
Recipient Characteristics					
Age (y)	61 (53, 66)	56 (46, 62)	60 (49.5, 64)	62 (55, 67)	<0.01*
Male sex (%)	451 (62%)	48 (65.8%)	81 (63.8%)	322 (61%)	0.66
Diagnosis					<0.01*
Cystic Fibrosis/Immunodeficiency	76 (10.4%)	12 (16.4%)	17 (13.4%)	47 (8.9%)	
Obstructive Lung disease	232 (31.9%)	35 (47.9%)	33 (26%)	164 (31.1%)	
Pulmonary Vascular disease	26 (3.6%)	3 (4.1%)	3 (2.4%)	20 (3.8%)	
Restrictive Lung disease	394 (54.1%)	23 (31.5%)	74 (58.3%)	297 (56.2%)	
Medical Condition					0.13
Not Hospitalized	562 (77.2%)	61 (83.6%)	89 (70.1%)	412 (78%)	
Hospitalized	65 (8.9%)	6 (8.2%)	17 (13.4%)	42 (8%)	
In ICU	101 (13.9%)	6 (8.2%)	21 (16.5%)	74 (14%)	
Functional Status					<0.01*
ADL With No Assistance	154 (21.5%)	36 (50.7%)	18 (14.4%)	100 (19.2%)	
ADL With Assistance	301 (42%)	24 (33.8%)	62 (49.6%)	215 (41.3%)	
Disabled/Hospitalized	261 (36.5%)	11 (15.5%)	45 (36%)	205 (39.4%)	
On Ventilator	44 (6%)	5 (6.8%)	13 (10.2%)	26 (4.9%)	0.08
LAS	39.1 (34.2, 51.7)	36 (33.2, 41.8)	42.8 (35, 59.5)	39.1 (34.3, 51.7)	<0.01*
PRA	0 (0, 0)	0 (0, 3)	0 (0, 2.5)	0 (0, 0)	<0.01*
Days on Waitlist	49 (14, 175)	138 (47, 368)	54 (12.5, 198)	44 (14, 138.5)	<0.01*
Previous ECMO/on ECMO	50 (6.9%)	4 (5.5%)	11 (8.7%)	35 (6.6%)	0.64
Donor Characteristics					
Age	39 (28, 48)	41 (29, 47)	39 (26.5, 49)	38 (28, 48)	0.90
Male sex (%)	433 (59.5%)	40 (54.8%)	90 (70.9%)	303 (57.4%)	0.02*
Smoking History	53 (7.4%)	12 (16.4%)	9 (7.1%)	32 (6.1%)	<0.01*
Anoxia Cause of Brain Injury	286 (39.3%)	24 (32.9%)	44 (34.6%)	218 (41.3%)	<0.01*
Donor Pulmonary Infection	436 (59.9%)	23 (31.5%)	70 (55.1%)	343 (65%)	< 0.01*
PaO2/FiO2 Ratio	416.1 ± 88.3	443.1 ± 84.2	416.5 ± 87.7	412.4 ± 88.6	0.03*
DCD Donor Lung Utilization (%)^A^	3.3%	1.3%	2.2%	5.0%	0.05*
Percentage of organ donors that are DCD^B^	15.1% (20,396/135,521)	9.8% (3,883/39,755)	13.9% (5,745/41,450)	19.8% (10,768/54,316)	0.04*
DCD Fraction of all Lung Transplants (%)	2.6%	1.1%	1.5%	4.2%	0.04*
Operative Characteristics and Outcomes					
Single Lung Transplant	168 (23.1%)	18 (24.7%)	46 (36.2%)	104 (19.7%)	<0.01*
Centers with DCD Lung transplant (% of all Lung Transplant Centers)	41 (51.3%)	14 (21.2%)	24 (33.8%)	38 (54.3%)	
Center DCD Volume	4 (2, 12)	3 (1, 6.75)	3 (1.75, 4.25)	10 (3.25, 18)	<0.01*
Ischemia Time (hours)	6.3 (5.1, 8.2)	5.6 (4.6, 6.6)	5.8 (4.7, 7.6)	6.5 (5.3, 8.7)	<0.01*
Length of Stay (days)	21 (14, 37)	17 (12, 29)	21 (14, 37)	22 (14, 38)	0.03*
Postop Dialysis	81 (11.1%)	8 (11%)	14 (11%)	59 (11.2%)	0.99

Data displayed as mean ± standard deviation (SD) median (interquartile range) for parametric or non-parametric continuous variables respectively and number (percent of total) for categorical variables. BMI, body mass index; GFR, glomerular filtration rate; ICU, intensive care unit; ADL, activities of daily living; LAS, lung allocation score; PRA, percent reactive antibodies; ECMO, extracorporeal membrane oxygenation. * indicates *p* < 0.05. ^A^ “DCD Donor Lung Utilization (%)” calculated as fraction of DCD donors where a lung was procured and transplanted divided by all DCD donors regardless of which organ was donated. ^B^ “Percentage of all Organ Donors that are DCD” calculated as all DCD donors regardless of which organ was donated divided by all organ donors (DBD and DCD).

Regarding DCD donors, median age did not differ by era (*p* = 0.90), however in era 3, donors were less likely to have a significant smoking history (*p* < 0.01) and more likely to have a clinically diagnosed infection (*p* < 0.01). Median donor PF ratio also decreased after era 1 (*p* = 0.03). Other donor characteristics were similar amongst all eras (Table 4, Supplementary Table S2). The fraction of all lung transplants using a DCD donor increased from 1.1% of donors in era 1, to 1.5% in era 2 and 4.2% in era 3 (*p* = 0.04). The fraction of all organ donors that are DCD, including those in whom the lungs were not used, has significantly increased from 9.8% in era 1, to 13.9% in era 2, and 19.8% in era 3 (*p* = 0.04). DCD lung donor utilization calculated as the fraction of all DCD donors where a lung was procured and transplanted also significantly increased from 1.3% in era 1, to 2.2% in era 2, and 5% in era 3 (*p* < 0.05). Regarding transplant characteristics, there was an increase in the total ischemic time from 5.6 h in Era 1 to 6.5 h in Era 3 (*p* < 0.01). There was also an increase in post-transplant length of stay from 17 days in Era 1 to 22 days in Era 3 (*p* = 0.03).

### Survival Analysis

**FIGURE 1:**
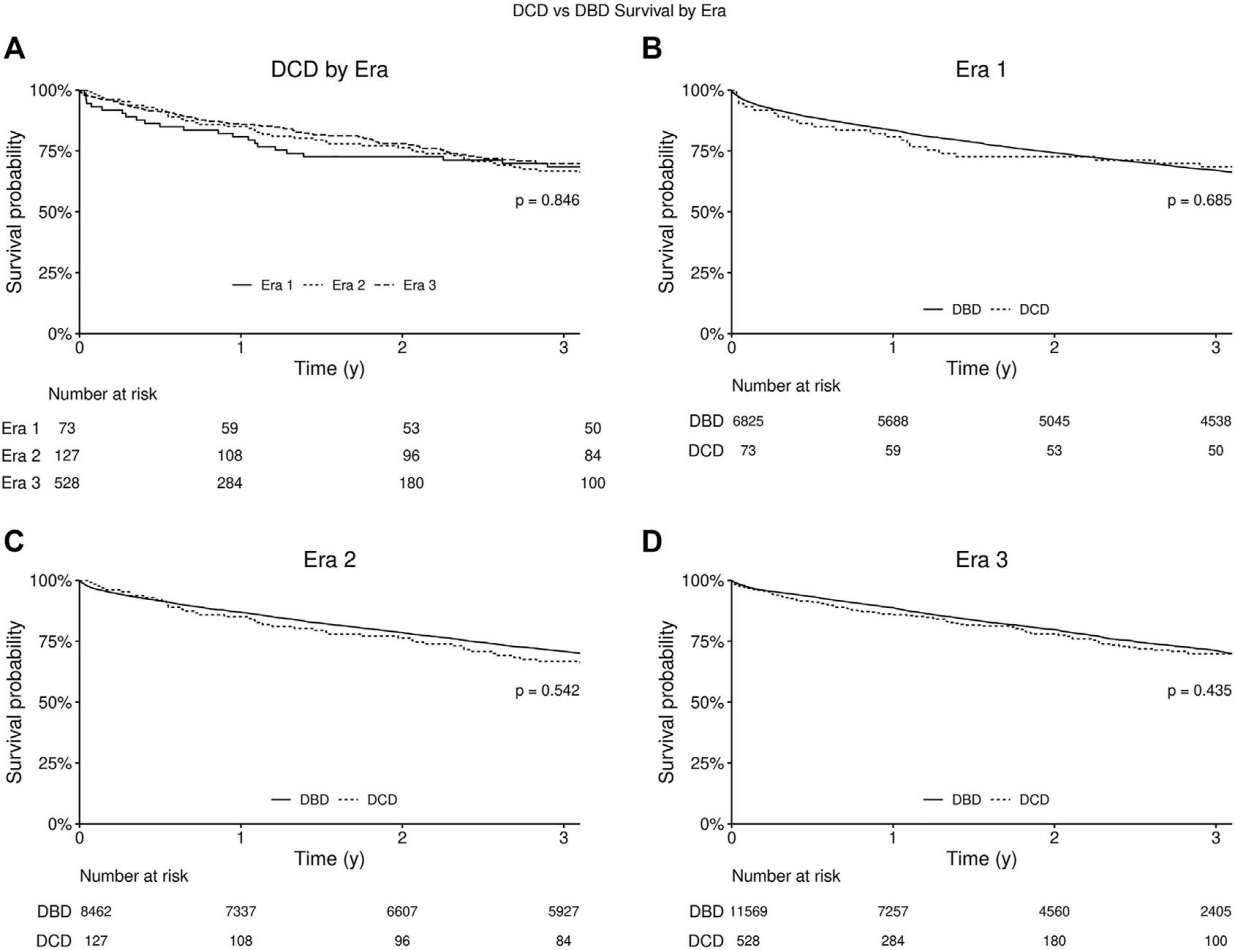
Kaplan-Meier survival curve for recipients of lungs from circulatory death donors (DCD) and brain dead donors (DBD). P-value for log-rank test comparing all three eras. **(A)** Kaplan-Meier survival curves for all eras from date of lung transplantation. **(B)** Kaplan-Meier survival curves for Era 1 (2005–2010). **(C)** Kaplan-Meier survival curves for Era 2 (2010–2015). **(D)** Kaplan-Meier survival curves for Era 3 (2015–2020).

**FIGURE 2:**
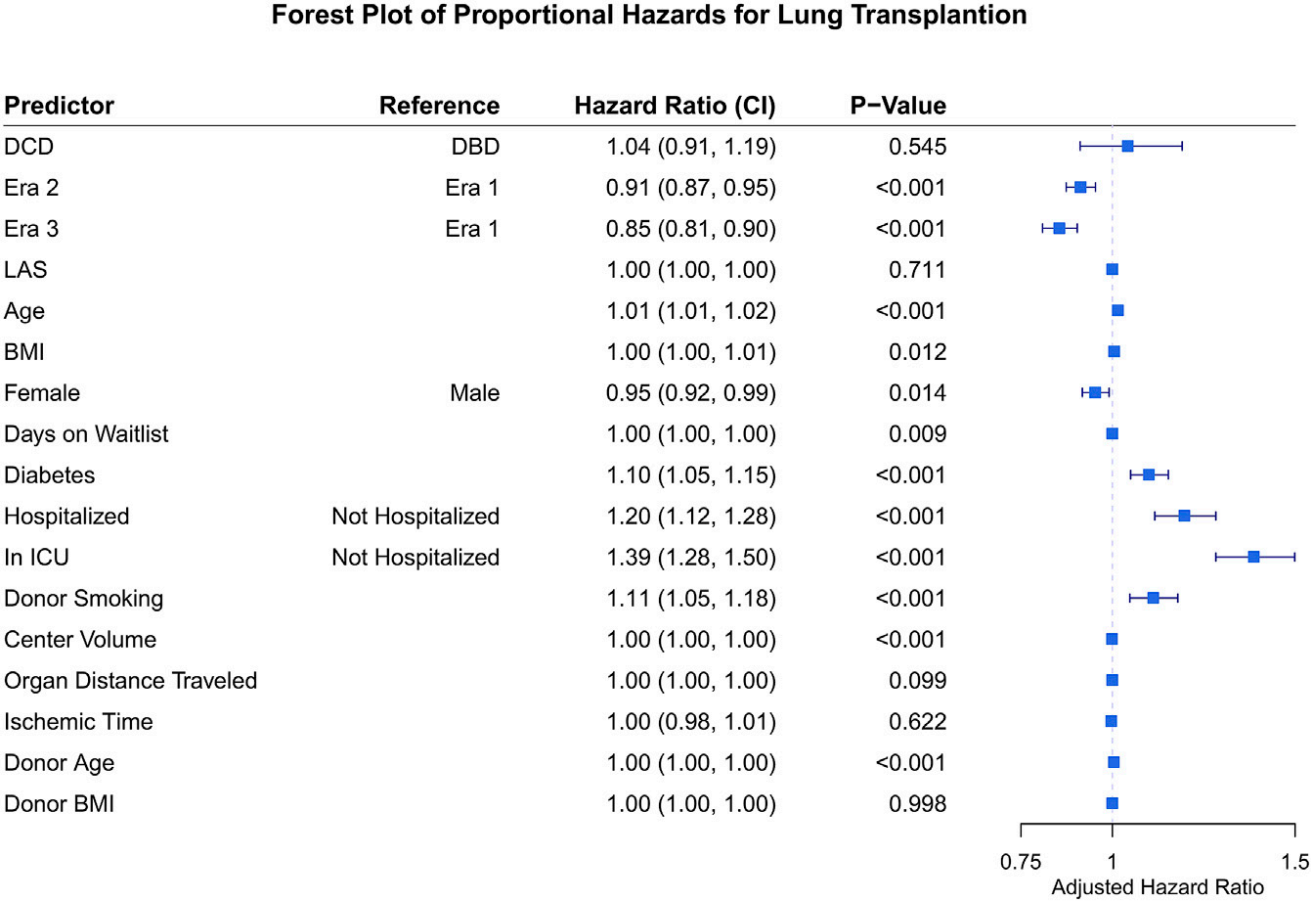
Forest plot for hazard ratio of death for covariates of interest following lung transplantation. DCD, donation after circulatory death; DBD, donation after brain death; LAS, lung allocation score; BMI, body mass index. ICU, intensive care unit.

With regard to survival there was no significant difference on unadjusted analysis between DCD and DBD organ recipients (Figure 1). Actuarial survival of DCD lung recipients at 3 years was 69.0% (95% CI: 65.1–73.3%) across all eras, 68.5% (CI: 95% 58.6–80.0%) in era 1, 66.8% (95% CI: 59.0–75.5%) in era 2 and 69.8% (95% CI: 65.6–75.4%) in era 3 (*p* = 0.85) (Figure 1A). There was no significant difference in survival between donor organs procured following brain death or circulatory death in all eras (Figures 1B–D). Cox proportional hazard model demonstrated usage of a DCD organ in lung transplantation was not associated with increased mortality (Hazard Ratio [HR] 1.04, 95% CI 0.91–1.19, *p* = 0.55), and transplant in more recent eras was associated with improved survival (era 2 HR 0.91, *p* < 0.01, and era 3 HR 0.85, *p* < 0.01) compared to era 1. Diabetes, poorer pre-operative health status, and donor smoking were all also associated with reduced survival in this model (Figure 2).

### Center Volume Trends and Geographic Variation in DCD Organ Use

**FIGURE 3:**
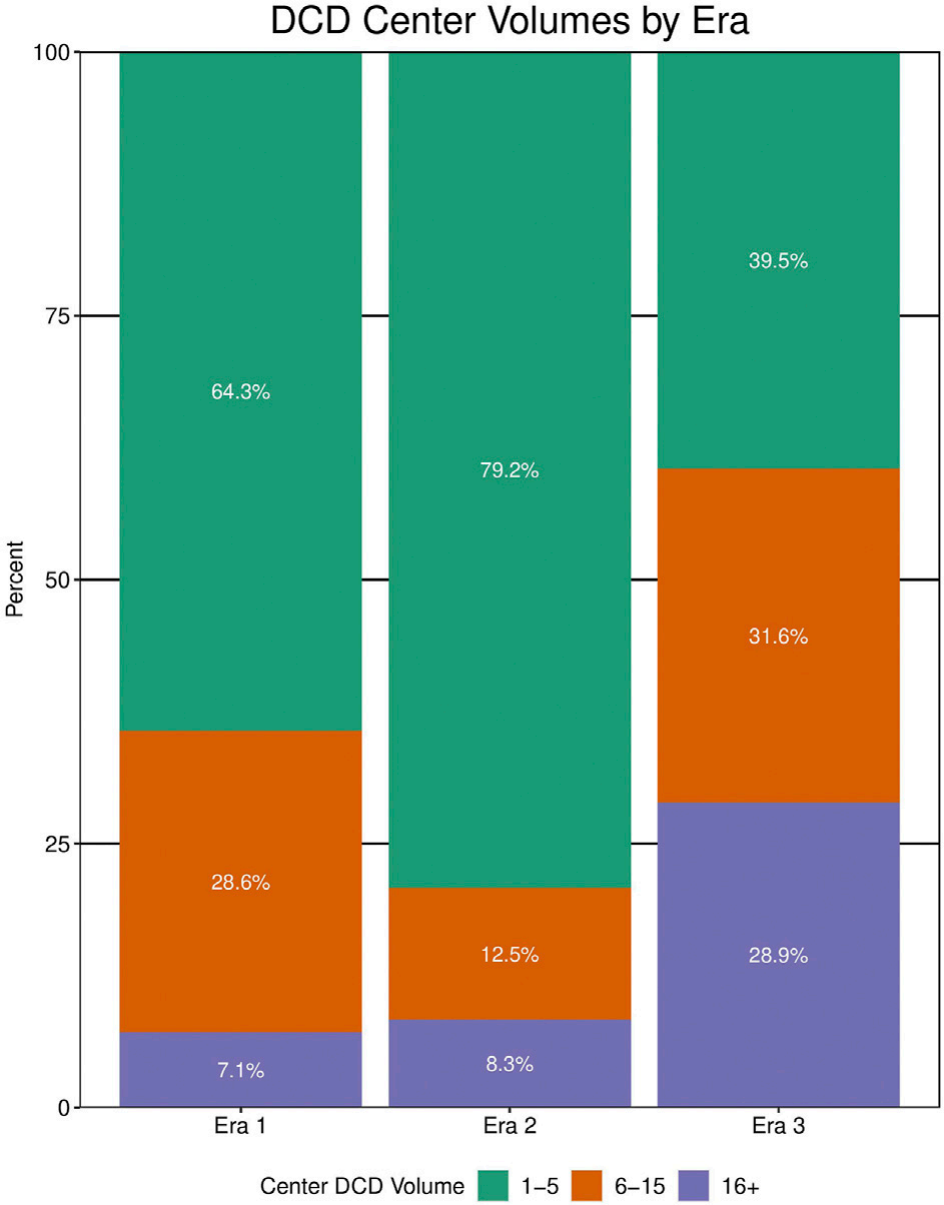
Center volume of donation after circulatory death (DCD) lung transplantation by era. Data for center volumes from any center performing >1 DCD lung transplant within that era.

**FIGURE 4:**
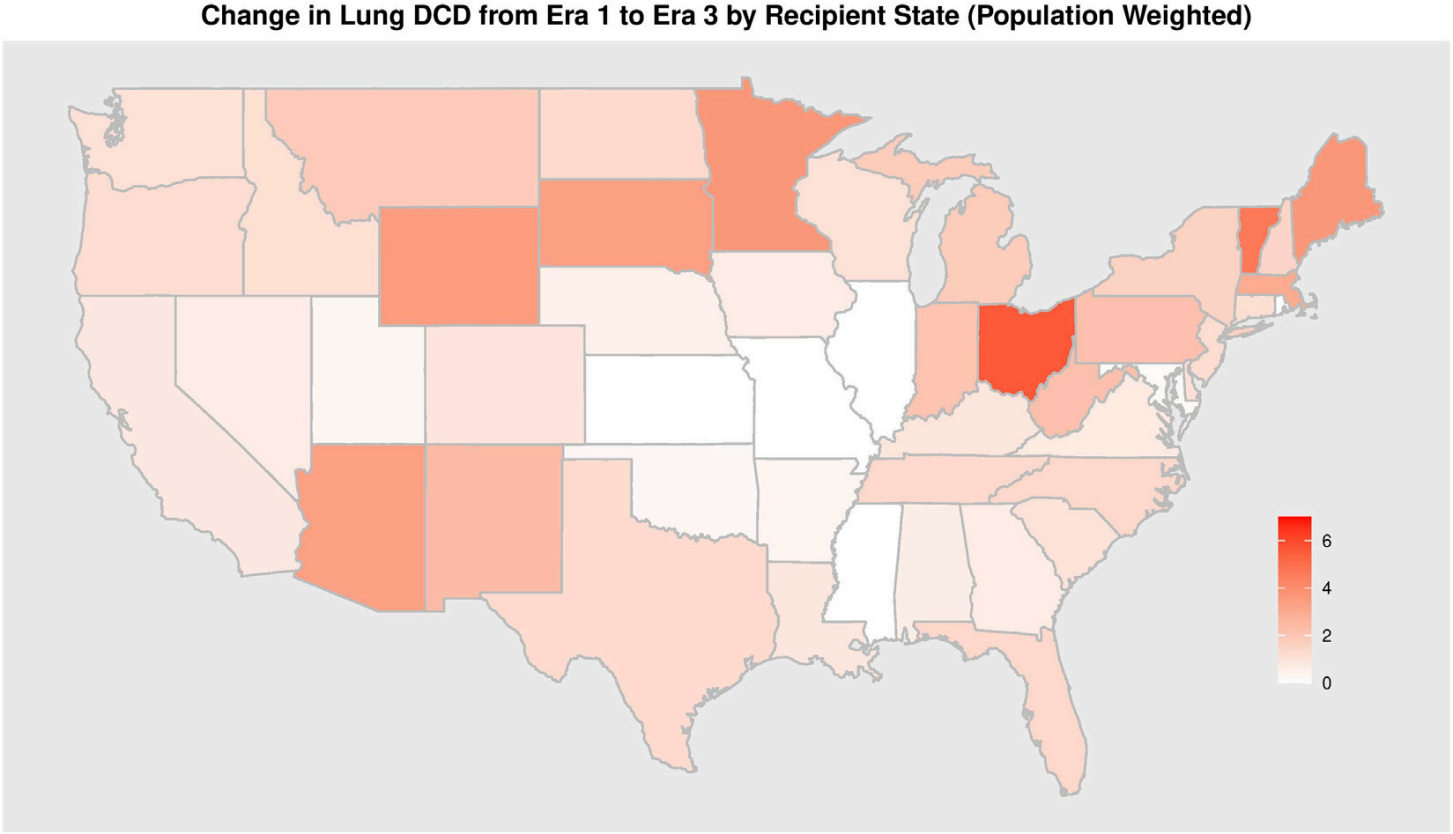
Change in Absolute Number of DCD Lung Transplantation from Era 1 to Era 3 by Recipient State per 1 million inhabitants.

41 different centers transplanted a lung from a DCD donor since 2005. The total number of centers utilizing DCD lungs for transplantation increased from 14 in era 1 to 24 in era 2 to 38 in era 3 (Table 4). Of all U.S. centers performing lung transplantation, 21.2% performed a DCD lung transplant in era 1, 33.8% in era 2, and 54.3% in era 3. Within centers that used DCD lungs, the total DCD lung volume was stable between era 1 and 2 before increasing in era 3 (Figure 3). Of all centers participating in DCD lung usage, in era 1 64.3% transplanted 1-5 DCD lungs, in era 2 this grew to 79.2%, and shrank to 39.5% in era 3. However, in era 3, 28.9% of centers transplanted >15 DCD lungs, compared to 7.1% in era 1. There was also geographic variation observed in DCD use over time (Figure 4; Supplementary Table S3). When weighted by population, Ohio increased the most from 1.2 DCD donors per million population (PMP) in era 1 to 6.8 DCD donors pmp in era 3, followed by Vermont and Minnesota. The largest absolute increase was observed in Ohio, which increased its use of DCD from 14 in era 1 to 80 in era 3. Other states with large increases were New York and Texas.

## Discussion

In this analysis we demonstrated that lung transplant recipients of DCD organs were older, more likely to be in the ICU or on ECMO pre-operatively and had shorter waitlist time compared to recipients of DBD lungs. However, DCD organs also had greater ischemic time, and recipients had a greater post-operative length of stay and use of dialysis. Despite these differences, DCD recipients continue to have similar survival to recipients of DBD lungs, on both unadjusted and adjusted survival analyses. There has been expansion in DCD use, but the overall number of DCD lungs used for lung transplantation remains low. Together these data characterize DCD lung characteristics and outcomes in the LAS era in the United States.

Recipient outcomes from DCD donors were equivalent to DBD donors and remained so throughout each era. Since era 1, the DCD recipient population became older and sicker, mirroring similar changes that have occurred in the lung transplant population as a whole (1). Waitlist time also decreased across each era and was lower in recipients of DCD lungs compared to DBD lungs. These are promising changes as they may represent an increasing acceptance of DCD lungs as a robust means to expand the donor pool and may demonstrate a greater overall sense of comfort in the use of DCD organs by transplant teams.(6, 9) Though DCD use has increased >7-fold since era 1, it still only comprises 4.2% of all lung transplants in the United States. Additionally, our data shows that donation after circulatory death comprises ∼20% of all organ donation (across all types of organ donation) in the United States. This suggests that DCD use for lung transplant has room for further growth. In the most recent era DCD organ use comprised only 4.2% of all lung transplants. This small percentage of usage relative to the number of DCD organs available represents an opportunity for lung transplant centers, particularly for those with longer waitlist times or increased waitlist mortality rates. Globally, the experience with DCD usage is different than in the United States (10). In a European survey of DCD use in lung transplantation, 1,381 DCD lung transplants were identified from 2008 to 2016 (11). This exceeds the 728 DCD lung transplants that we identified in the United States from 2005 to 2020. Moreover, in 2016 the same European state consortium reported 218 controlled DCD lung transplants, and 15 uncontrolled DCD lung transplants, out of 2,549 total lung transplants. DCD lungs composed 9.3% of all lung transplants that year, compared to 4.2% in the US in the most recent era. In certain countries such as Australia, United Kingdom, and Netherlands, the use of DCD for lung transplantation is 30–50% (11, 12). This could be due to greater standardization and more explicit regulations around what is and is not allowed during procurement (some European states allow pre-mortem interventions such as administration of heparin or cannulation). Or it could be due to differences in consenting processes (i.e., opt-in or out-out consent for organ donation after death) (13).

Several barriers exist that may slow the growth of DCD use in the United States. Utilization of DCD lungs requires transplant programs to view the organs as viable and equivalent to traditional DBD organs. This study adds to the growing foundation of literature supporting this concept. Our data indicate that over the past 15 years, the proportion of lung transplant centers using DCD lungs has increased from 21.2% to 54.3%. Retrospective studies and meta-analyses have previously shown inconsistent results regarding long-term survival and operative complications using DCD lungs, possibly slowing the rate of DCD adoption by new centers. A 2020 systematic review and meta-analysis found no difference in 1-year survival or PGD between DCD and DBD organs, but observed an increase in airway complications and a reduction in 5-year survival in DCD organs (14). However, due to the relatively small overall DCD cohort size and single-center nature of most of the included studies, the study mentions a high likelihood of allocation bias. Two additional meta-analyses found no difference in 1-year survival between DBD and DCD lungs.(4, 15) In a database analysis of the International Society for Heart and Lung Transplantation (ISHLT) registry using unadjusted and multivariable analyses, there was no difference in 5-year survival between DBD and DCD organs, though a survival benefit was associated with era of transplant (2003–2009 vs. 2010–2016) (16). Similarly our analysis using 15 years of data and over 27,000 lung transplant cases in the United States, did not demonstrate a survival difference between DBD and DCD lungs, but found a survival benefit associated with more recent era of transplant. In addition to uncertainty about graft viability and survival (17), transplantation teams prefer to have intraoperative organ assessment prior to transplantation. Prior to a DCD procurement there is no opportunity for determination of intraoperative PF ratio, something commonly performed in DBD donors following lung recruitment where the chest has already been opened. Additionally, as the patient is not deceased, the workup (i.e., scans, bronchoscopy, etc.) of the donor may be less comprehensive than DBD donors. Our data suggests teams used a more conservative donor PF ratio in earlier eras, as DCD donor PF ratio decreased from era 1 (443) to era 3 (412). EVLP, however, provides a technique allowing for pre-implantation assessment of donor lung allografts that may help alleviate concerns of organ functional assessment prior to transplantation (18). SRTR began collecting data on organ perfusion prior to transplantation on 2/28/2018. Our data indicate that EVLP use in era 3 was 27.3% in DCD lungs compared to 3.8% of DBD lungs. This suggests practitioners are preferentially utilizing EVLP for assessment of DCD lungs. As availability of EVLP continues to increase (including third party services that can be contracted for EVLP use), this may help to alleviate further center barriers to DCD organ adoption. As additional evidence accumulates around the efficacy of DCD lungs, the volume of DCD lungs should continue to expand in the pursuit of reducing waitlist time and mortality.

DCD lung transplantation has expanded in its utilization, however, not uniformly across all centers. Over time, an increasing number of centers have elected to use DCD lungs and the DCD volume within those centers has increased. Despite this increase, only about 50% of all centers have used a DCD organ for lung transplantation. From era 1 to era 2, the number of centers utilizing a DCD lung increased, but the median DCD volume at those centers remained unchanged. However, as DCD expansion continued into era 3, there was an increase in both the number of centers using DCD and median DCD volume. This may reflect a transition in DCD lung transplantation from an experimental novelty to a real avenue for growth in transplant volume. We also identified geographic variation in DCD use. We observed that DCD use constitutes a larger percentage (∼15%) of all lung recipients from certain states and reliance on DCD organs for lung transplant is generally concentrated in the northern portion of the United States (Supplementary Figures S1, S2). A recent analysis of DCD usage by OPO confirms a similar geographic pattern of use (19). Our analysis helps to provide more granularity to these previously published findings as well as add context through an analysis of donor and recipient profiles. Several potential elements may determine why certain states and centers increasingly rely on DCD lungs. During procurement for a DCD organ, the patient is extubated and a pre-determined time is allotted for declaration of death. This process of how death is declared and the time frame for progression to death varies by center and jurisdiction (20). There is no universal protocol for sedation or allotted post-extubation time, and variations in these factors (within an OPO or hospital) has the potential to impact whether a donor organ can be procured (21). Other differences in logistical management by centers and OPOs create variability (22). For example, rules regulating when surgical teams are allowed in the OR, when heparin is administered, how long procuring teams wait for declaration of death, and protocols governing comfort care surrounding withdrawal commonly differ amongst hospitals and OPOs. Additional factors include an OPO or donor hospital’s willingness to perform recruitment maneuvers, bronchoscopies, and CT scans on potential DCD donors. Lastly, resource and labor availability also affect a center’s likelihood of sending a team for a DCD lung procurement, as it has a potentially lower chance of conversion to transplant than a DBD procurement. The use of local procurement teams (as is commonly done for kidney procurement) could help address this issue, though the importance of intraoperative assessment of lungs such as compliance, unlike kidneys, may limit centers enthusiasm. In comparison to protocols and consensus statements for organ procurement following DBD, DCD organ procurement is less standardized (23). Given the variability observed in the United States we propose working towards more-refined consensus statements and idealized protocols for DCD lung procurement, which may impact increased utilization.

### Limitations

There are some limitations to our findings. Our large dataset is multi-center and retrospective and is therefore subject to information and selection bias. Longer survival data are necessary to compare to DBD and DCD lungs (17), especially for the most recent era where a larger number of DCD transplants were performed. Furthermore, we did not analyze the association of DCD or DBD organs with chronic lung allograft dysfunction, nor we did not investigate the cause of DCD organs rejected for transplantation (24). Additionally, geographic analysis was conducted at the recipient level, not where donor procurement took place or by implanting institution. Our data is also subject to selection bias as we are only analyzing DCD organs that were transplanted and not assessing organs that were deemed unsuitable for transplantation following attempted procurement. In analyzing the PaO2/FiO2 ratio, UNOS does not specify the timing of sample collection. It is possible samples are taken from the ICU before procurement or even in the operating room after full lung recruitment. Finally, we do not have EVLP use data prior to 2018, due to lack of data in the STAR file, which limits better characterization of DCD organs prior to transplantation.

## Conclusion

In summary, use of DCD lungs has increased over time, with similar long-term survival compared to DBD lungs despite higher ischemic time. Continued increases in DCD volume will help expand the lung donor pool, particularly for recipients with limitations on size and antibody profile. Given the heterogeneous geographic distribution in DCD utilization further investigation into limiting factors for utilization is warranted and may justify protocol standardization for these donors.

## Data Availability

The original contributions presented in the study are included in the article/Supplementary Material, further inquiries can be directed to the corresponding author.

## Abbreviations

ANOVA, analysis of variance; DBD, donation after brain death; DCD, donation after circulatory death; EVLP, ex vivo lung perfusion; ISHLT, international society for heart and lung transplantation; LAS, lung allocation score; PF, PaO2/FiO2; PGD, primary graft dysfunction; PMP, per million in population; OPO, organ procurement organization; TRR, transplant recipient registration form; UNOS/OPTN, united network for organ sharing/organ procurement and transplant network.
