# Supplementary material for: Trends in Donation After Circulatory Death in Lung Transplantation in the United States: Impact Of Era
Source: Transpl Int. 2022 Apr 4;35:10172. doi: 10.3389/ti.2022.10172 (PMC9013720; doi:10.3389/ti.2022.10172)

SUPPLEMENTAL TABLES AND FIGURES

Supplemental Table 1. Missingness and Normality (Kolmogorov-Smirnov test) for all variables

| **Variable** | **P-value** | **Missing data (% total)** |
| --- | --- | --- |
| Age | < 0.001 | 0 |
| Sex |  | 0 |
| Ethnicity |  | 0 |
| BMI | 0.199 | 0 |
| Former Smoker |  | 0.82 |
| Diabetes |  | 0 |
| Creatinine (mg/dL) | < 0.001 | 0 |
| GFR (mL/min/1.73m2) | < 0.001 | 0.13 |
| Pre-operative Dialysis |  | 72.52 |
| Diagnosis |  | 0 |
| Blood Group |  | 0 |
| Medical Condition |  | 0 |
| Functional Status |  | 1.64 |
| On Ventilator |  | 0 |
| LAS | < 0.001 | 0 |
| PRA | < 0.001 | 0 |
| Days on Waitlist | < 0.001 | 0 |
| Previous ECMO/on ECMO |  | 0 |
| Age | 0.001 | 0 |
| Sex |  | 0 |
| Ethnicity |  | 0 |
| BMI | < 0.001 | 0.13 |
| Coronary Artery Disease |  | 0 |
| Smoking History |  | 1.09 |
| Recent Cocaine Use |  | 0.96 |
| Diabetes |  | 0.41 |
| Hypertension |  | 0.68 |
| Donor Cause of Death |  | 0 |
| Donor Bloodstream Infection |  | 0 |
| Donor Clinical Infection |  | 0.54 |
| Donor Pulmonary Infection |  | 0 |
| PaO2/FiO2 Ratio | 0.22 | 3.15 |
| Type of Transplant |  | 0 |
| Distance Traveled | < 0.001 | 0 |
| Ischemic Time | < 0.001 | 1.92 |
| Length of Stay (Days) | < 0.001 | 2.06 |
| Postop Dialysis |  | 0 |
| Postop Stroke |  | 0.68 |
| Postop Dehiscence |  | 0.96 |
| In Hospital Mortality |  | 2.47 |
| Acute Rejection Before Discharge |  | 0 |
| Rejection Treatment Within One Year |  | 31.31 |
| EVLP |  | 55.08 |

BMI, body mass index; GFR, glomerular filtration rate; LAS, lung allocation score; PRA, percent reactive antibodies; ECMO, extracorporeal membrane oxygenation; EVLP, ex vivo lung perfusion

Supplemental Table 2. DCD Characteristics by Era for all Variables

| **Variable** | **Overall** | **Era 1** | **Era 2** | **Era 3** | **P-value** |
| --- | --- | --- | --- | --- | --- |
| Date Range |  | 5-1-2005 to 4-30-2010 | 5-1-10 to 4-30-2015 | 5-1-2015 to 4-30-2020 |  |
| Cohort Size |  | 73 | 127 | 528 |  |
| **Recipient Demographics and Baseline Characteristics** | | |  |  |  |
| Age | 61 (53, 66) | 56 (46, 62) | 60 (49.5, 64) | 62 (55, 67) | < 0.01 |
| Sex |  |  |  |  | 0.66 |
| Male | 451 (62%) | 48 (65.8%) | 81 (63.8%) | 322 (61%) |  |
| Female | 277 (38%) | 25 (34.2%) | 46 (36.2%) | 206 (39%) |  |
| Ethnicity |  |  |  |  | 0.23 |
| White | 616 (84.6%) | 65 (89%) | 112 (88.2%) | 439 (83.1%) |  |
| Black | 65 (8.9%) | 6 (8.2%) | 11 (8.7%) | 48 (9.1%) |  |
| Other | 47 (6.5%) | 2 (2.7%) | 4 (3.1%) | 41 (7.8%) |  |
| BMI | 25.4 (22, 28.7) | 24.2 (20.4, 27.9) | 24.8 (21.9, 28.6) | 25.6 (22.3, 28.7) | 0.11 |
| Former Smoker |  |  |  |  | 0.62 |
| No | 290 (40.2%) | 24 (35.3%) | 49 (38.9%) | 217 (41.1%) |  |
| Yes | 432 (59.8%) | 44 (64.7%) | 77 (61.1%) | 311 (58.9%) |  |
| Diabetes |  |  |  |  | 0.11 |
| No | 587 (80.6%) | 61 (83.6%) | 94 (74%) | 432 (81.8%) |  |
| Yes | 141 (19.4%) | 12 (16.4%) | 33 (26%) | 96 (18.2%) |  |
| Creatinine (mg/dL) | 0.8 (0.7, 1) | 0.8 (0.7, 0.9) | 0.8 (0.6, 1) | 0.8 (0.7, 1) | 0.62 |
| GFR (mL/min/1.73m2) | 96.1 (72.8, 122.1) | 98.9 (75.6, 127) | 100.2 (74.5, 128.9) | 94.8 (72.3, 121.5) | 0.38 |
| Pre-operative Dialysis |  |  |  |  |  |
| No | 199 (99.5%) | 64 (100%) | 109 (100%) | 26 (96.3%) |  |
| Yes | 1 (0.5%) | 0 (0%) | 0 (0%) | 1 (3.7%) |  |
| Diagnosis |  |  |  |  | 0.00 |
| Cystic Fibrosis/ Immunodeficiency | 76 (10.4%) | 12 (16.4%) | 17 (13.4%) | 47 (8.9%) |  |
| Obstructive Lung Disease | 232 (31.9%) | 35 (47.9%) | 33 (26%) | 164 (31.1%) |  |
| Pulmonary Vascular Disease | 26 (3.6%) | 3 (4.1%) | 3 (2.4%) | 20 (3.8%) |  |
| Restrictive Lung Disease | 394 (54.1%) | 23 (31.5%) | 74 (58.3%) | 297 (56.2%) |  |
| Blood Group |  |  |  |  | 0.69 |
| A | 303 (41.6%) | 29 (39.7%) | 49 (38.6%) | 225 (42.6%) |  |
| B | 68 (9.3%) | 6 (8.2%) | 12 (9.4%) | 50 (9.5%) |  |
| AB | 19 (2.6%) | 0 (0%) | 3 (2.4%) | 16 (3%) |  |
| O | 338 (46.4%) | 38 (52.1%) | 63 (49.6%) | 237 (44.9%) |  |
| Medical Condition |  |  |  |  | 0.13 |
| Not Hospitalized | 562 (77.2%) | 61 (83.6%) | 89 (70.1%) | 412 (78%) |  |
| Hospitalized | 65 (8.9%) | 6 (8.2%) | 17 (13.4%) | 42 (8%) |  |
| In ICU | 101 (13.9%) | 6 (8.2%) | 21 (16.5%) | 74 (14%) |  |
| Functional Status |  |  |  |  | < 0.01 |
| ADL With No Assistance | 154 (21.5%) | 36 (50.7%) | 18 (14.4%) | 100 (19.2%) |  |
| ADL With Assistance | 301 (42%) | 24 (33.8%) | 62 (49.6%) | 215 (41.3%) |  |
| Disabled/Hospitalized | 261 (36.5%) | 11 (15.5%) | 45 (36%) | 205 (39.4%) |  |
| On Ventilator |  |  |  |  | 0.08 |
| No | 684 (94%) | 68 (93.2%) | 114 (89.8%) | 502 (95.1%) |  |
| Yes | 44 (6%) | 5 (6.8%) | 13 (10.2%) | 26 (4.9%) |  |
| LAS | 39.1 (34.2, 51.7) | 36 (33.2, 41.8) | 42.8 (35, 59.5) | 39.1 (34.3, 51.7) | < 0.01 |
| PRA | 0 (0, 0) | 0 (0, 3) | 0 (0, 2.5) | 0 (0, 0) | 0.01 |
| Days on Waitlist | 49 (14, 175) | 138 (47, 368) | 54 (12.5, 198) | 44 (14, 138.5) | < 0.01 |
| Previous ECMO/on ECMO |  |  |  |  | 0.64 |
| No ECMO | 678 (93.1%) | 69 (94.5%) | 116 (91.3%) | 493 (93.4%) |  |
| ECMO | 50 (6.9%) | 4 (5.5%) | 11 (8.7%) | 35 (6.6%) |  |
| **Donor Characteristics** | |  |  |  |  |
| Age | 39 (28, 48) | 41 (29, 47) | 39 (26.5, 49) | 38 (28, 48) | 0.90 |
| Sex |  |  |  |  | 0.02 |
| Male | 433 (59.5%) | 40 (54.8%) | 90 (70.9%) | 303 (57.4%) |  |
| Female | 295 (40.5%) | 33 (45.2%) | 37 (29.1%) | 225 (42.6%) |  |
| Ethnicity |  |  |  |  | 0.44 |
| White | 595 (81.7%) | 66 (90.4%) | 104 (81.9%) | 425 (80.5%) |  |
| Black | 49 (6.7%) | 3 (4.1%) | 10 (7.9%) | 36 (6.8%) |  |
| Hispanic | 57 (7.8%) | 3 (4.1%) | 11 (8.7%) | 43 (8.1%) |  |
| Asian | 18 (2.5%) | 0 (0%) | 1 (0.8%) | 17 (3.2%) |  |
| Other | 9 (1.2%) | 1 (1.4%) | 1 (0.8%) | 7 (1.3%) |  |
| BMI | 26.3 (23, 31) | 25.3 (22.8, 29.4) | 25.9 (23.6, 30.1) | 26.5 (23, 31.2) | 0.72 |
| Coronary Artery Disease |  |  |  |  | 0.58 |
| No Documented Coronary Disease | 720 (98.9%) | 73 (100%) | 125 (98.4%) | 522 (98.9%) |  |
| Documented Coronary Disease | 8 (1.1%) | 0 (0%) | 2 (1.6%) | 6 (1.1%) |  |
| Smoking History |  |  |  |  | 0.01 |
| No | 667 (92.6%) | 61 (83.6%) | 117 (92.9%) | 489 (93.9%) |  |
| Yes | 53 (7.4%) | 12 (16.4%) | 9 (7.1%) | 32 (6.1%) |  |
| Recent Cocaine Use |  |  |  |  | 0.02 |
| No | 575 (79.8%) | 58 (80.6%) | 110 (88.7%) | 407 (77.5%) |  |
| Yes | 146 (20.2%) | 14 (19.4%) | 14 (11.3%) | 118 (22.5%) |  |
| Diabetes |  |  |  |  | 0.82 |
| No | 674 (93%) | 69 (94.5%) | 117 (92.1%) | 488 (93%) |  |
| Yes | 51 (7%) | 4 (5.5%) | 10 (7.9%) | 37 (7%) |  |
| Hypertension |  |  |  |  | 0.39 |
| No | 538 (74.4%) | 57 (78.1%) | 99 (78%) | 382 (73%) |  |
| Yes | 185 (25.6%) | 16 (21.9%) | 28 (22%) | 141 (27%) |  |
| Inciting Event Leading to Donation |  |  |  |  | 0.01 |
| Anoxia | 286 (39.3%) | 24 (32.9%) | 44 (34.6%) | 218 (41.3%) |  |
| CVA | 199 (27.3%) | 17 (23.3%) | 26 (20.5%) | 156 (29.5%) |  |
| Head Trauma | 227 (31.2%) | 29 (39.7%) | 53 (41.7%) | 145 (27.5%) |  |
| CNS Tumor | 1 (0.1%) | 0 (0%) | 1 (0.8%) | 0 (0%) |  |
| Other | 15 (2.1%) | 3 (4.1%) | 3 (2.4%) | 9 (1.7%) |  |
| Donor Bloodstream Infection |  |  |  |  | 0.09 |
| No | 671 (92.2%) | 71 (97.3%) | 120 (94.5%) | 480 (90.9%) |  |
| Yes | 57 (7.8%) | 2 (2.7%) | 7 (5.5%) | 48 (9.1%) |  |
| Donor Clinical Infection |  |  |  |  | < 0.01 |
| No | 231 (31.9%) | 45 (62.5%) | 46 (36.2%) | 140 (26.7%) |  |
| Yes | 493 (68.1%) | 27 (37.5%) | 81 (63.8%) | 385 (73.3%) |  |
| Donor Pulmonary Infection |  |  |  |  | < 0.01 |
| No | 292 (40.1%) | 50 (68.5%) | 57 (44.9%) | 185 (35%) |  |
| Yes | 436 (59.9%) | 23 (31.5%) | 70 (55.1%) | 343 (65%) |  |
| PaO2/FiO2 Ratio | 423 (360, 481) | 452 (384.8, 505.8) | 420 (361.5, 481) | 423 (359.4, 475) | 0.03 |
| Lung DCD Utilization^A^ | 2.6% | 1.1% | 1.5% | 4.2% | <0.05 |
| Percentage of all Organ Donors that are DCD^B^ | 15.1% | 9.8% | 13.9% | 19.8% | <0.04 |
| **Operative Characteristics and Postoperative Outcomes** | | |  |  |  |
| Type of Transplant |  |  |  |  | < 0.01 |
| Bilateral | 560 (76.9%) | 55 (75.3%) | 81 (63.8%) | 424 (80.3%) |  |
| Single | 168 (23.1%) | 18 (24.7%) | 46 (36.2%) | 104 (19.7%) |  |
| Centers Performing DCD Lung Transplant (% of all Lung Transplant Centers) | 41 (51.3%) | 14 (21.2%) | 24 (33.8%) | 38 (54.3%) |  |
| Center Volume | 4 (2, 12) | 3 (1, 6.75) | 3 (1.75, 4.25) | 10 (3.25, 18) | <0.01 |
| Distance Traveled | 113.5 (16, 325.2) | 143 (26, 379) | 107 (7, 380) | 111.5 (17, 296.8) | 0.58 |
| Ischemic Time | 6.3 (5.1, 8.2) | 5.6 (4.6, 6.6) | 5.8 (4.7, 7.6) | 6.5 (5.3, 8.7) | < 0.01 |
| Length of Stay (Days) | 21 (14, 37) | 17 (12, 29) | 21 (14, 37) | 22 (14, 38) | 0.03 |
| Postop Dialysis |  |  |  |  | 1.00 |
| No Dialysis | 647 (88.9%) | 65 (89%) | 113 (89%) | 469 (88.8%) |  |
| Dialysis | 81 (11.1%) | 8 (11%) | 14 (11%) | 59 (11.2%) |  |
| Postop Stroke |  |  |  |  | 0.89 |
| No Stroke | 708 (97.9%) | 71 (98.6%) | 122 (97.6%) | 515 (97.9%) |  |
| Stroke | 15 (2.1%) | 1 (1.4%) | 3 (2.4%) | 11 (2.1%) |  |
| Postop Dehiscence |  |  |  |  | 0.63 |
| No Airway Dehiscence | 705 (97.8%) | 69 (97.2%) | 121 (96.8%) | 515 (98.1%) |  |
| Airway Dehiscence | 16 (2.2%) | 2 (2.8%) | 4 (3.2%) | 10 (1.9%) |  |
| In Hospital Mortality |  |  |  |  | 0.59 |
| No | 668 (94.1%) | 66 (91.7%) | 120 (95.2%) | 482 (94.1%) |  |
| Yes | 42 (5.9%) | 6 (8.3%) | 6 (4.8%) | 30 (5.9%) |  |
| Acute Rejection Before Discharge |  |  |  |  | 0.13 |
| Yes & Treated with Immunosuppressant | 73 (10%) | 9 (12.3%) | 17 (13.4%) | 47 (8.9%) |  |
| Yes & Not Treated with Immunosuppressant | 10 (1.4%) | 0 (0%) | 4 (3.1%) | 6 (1.1%) |  |
| No | 645 (88.6%) | 64 (87.7%) | 106 (83.5%) | 475 (90%) |  |
| Rejection Treatment Within One Year |  |  |  |  | 0.03 |
| No | 357 (71.4%) | 37 (64.9%) | 66 (62.9%) | 254 (75.1%) |  |
| Yes | 143 (28.6%) | 20 (35.1%) | 39 (37.1%) | 84 (24.9%) |  |
| EVLP |  |  |  |  |  |
| No | 238 (72.8%) | - | - | 237 (72.7%) |  |
| Yes | 89 (27.2%) | - | - | 89 (27.3%) |  |

Data displayed as mean ± standard deviation (SD) median (interquartile range) for parametric or non-parametric continuous variables respectively and number (percent of total) for categorical variables. BMI, body mass index; GFR, glomerular filtration rate; ICU, intensive care unit; ADL, activities of daily living; LAS, lung allocation score; PRA, percent reactive antibodies; ECMO, extracorporeal membrane oxygenation; CVA, cerebrovascular accident; CNS, central nervous system; EVLP, ex vivo lung perfusion. ^A^ “DCD Donor Lung Utilization (%)” calculated as fraction of DCD donors where a lung was procured and transplanted divided by all DCD donors regardless of which organ was donated. ^B^ “Percentage of all Organ Donors that are DCD” calculated as all DCD donors regardless of which organ was donated divided by all organ donors (DBD and DCD).

Supplemental Table 3. DCD use in each state by era. State for each DCD donor was collected using recipient state of origin.

| **State** | **Era 1** | **Era 2** | **Era 3** | **Total** |
| --- | --- | --- | --- | --- |
| Alabama | 0 | 2 | 3 | 5 |
| Arizona | 0 | 3 | 24 | 27 |
| Arkansas | 1 | 0 | 2 | 3 |
| California | 7 | 4 | 37 | 48 |
| Colorado | 0 | 0 | 5 | 5 |
| Connecticut | 1 | 2 | 5 | 8 |
| Delaware | 0 | 0 | 1 | 1 |
| District Of Columbia | 0 | 0 | 1 | 1 |
| Florida | 1 | 2 | 30 | 33 |
| Georgia | 0 | 2 | 7 | 9 |
| Idaho | 0 | 0 | 2 | 2 |
| Illinois | 10 | 3 | 7 | 20 |
| Indiana | 2 | 2 | 16 | 20 |
| Iowa | 1 | 0 | 3 | 4 |
| Kansas | 0 | 0 | 0 | 0 |
| Kentucky | 2 | 0 | 6 | 8 |
| Louisiana | 0 | 2 | 4 | 6 |
| Maine | 0 | 0 | 5 | 5 |
| Maryland | 0 | 3 | 1 | 4 |
| Massachusetts | 0 | 3 | 21 | 24 |
| Michigan | 5 | 5 | 23 | 33 |
| Minnesota | 0 | 11 | 21 | 32 |
| Mississippi | 0 | 0 | 0 | 0 |
| Missouri | 1 | 3 | 0 | 4 |
| Montana | 0 | 0 | 2 | 2 |
| Nebraska | 0 | 3 | 1 | 4 |
| Nevada | 0 | 0 | 2 | 2 |
| New Hampshire | 0 | 1 | 2 | 3 |
| New Jersey | 1 | 4 | 12 | 17 |
| New Mexico | 1 | 0 | 6 | 7 |
| New York | 14 | 18 | 46 | 78 |
| North Carolina | 0 | 7 | 14 | 21 |
| North Dakota | 0 | 0 | 1 | 1 |
| Ohio | 14 | 23 | 80 | 117 |
| Oklahoma | 0 | 0 | 1 | 1 |
| Oregon | 0 | 0 | 5 | 5 |
| Pennsylvania | 5 | 4 | 34 | 43 |
| Rhode Island | 0 | 1 | 0 | 1 |
| South Carolina | 0 | 1 | 5 | 6 |
| South Dakota | 0 | 2 | 3 | 5 |
| Tennessee | 0 | 0 | 9 | 9 |
| Texas | 0 | 2 | 36 | 38 |
| Utah | 0 | 0 | 1 | 1 |
| Vermont | 0 | 2 | 3 | 5 |
| Virginia | 0 | 1 | 6 | 7 |
| Washington | 0 | 0 | 8 | 8 |
| West Virginia | 2 | 0 | 6 | 8 |
| Wisconsin | 5 | 10 | 11 | 26 |
| Wyoming | 0 | 0 | 2 | 2 |

Supplemental Figure 1. DCD percent of all lung donors in each state by era.


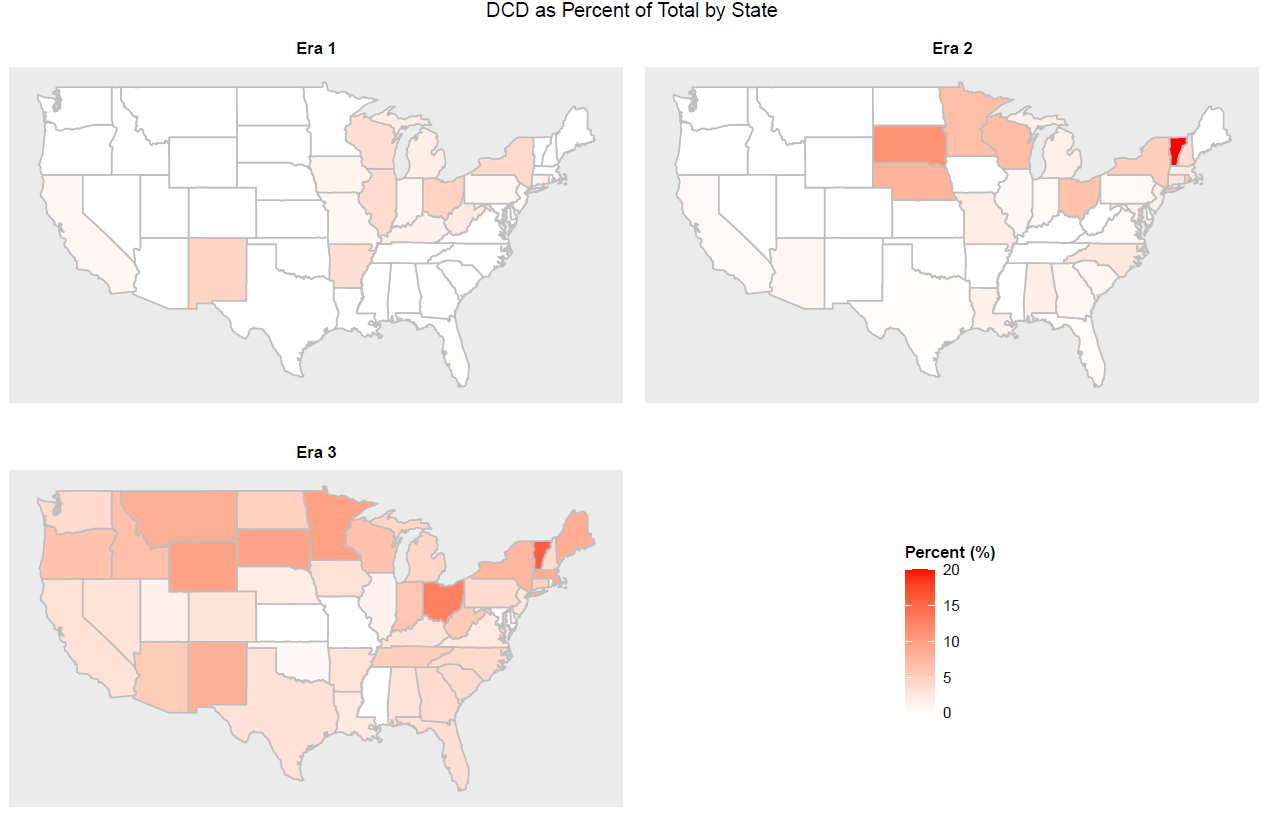


Supplemental Figure 2. Change in DCD fraction of all lungs transplants by recipient state.


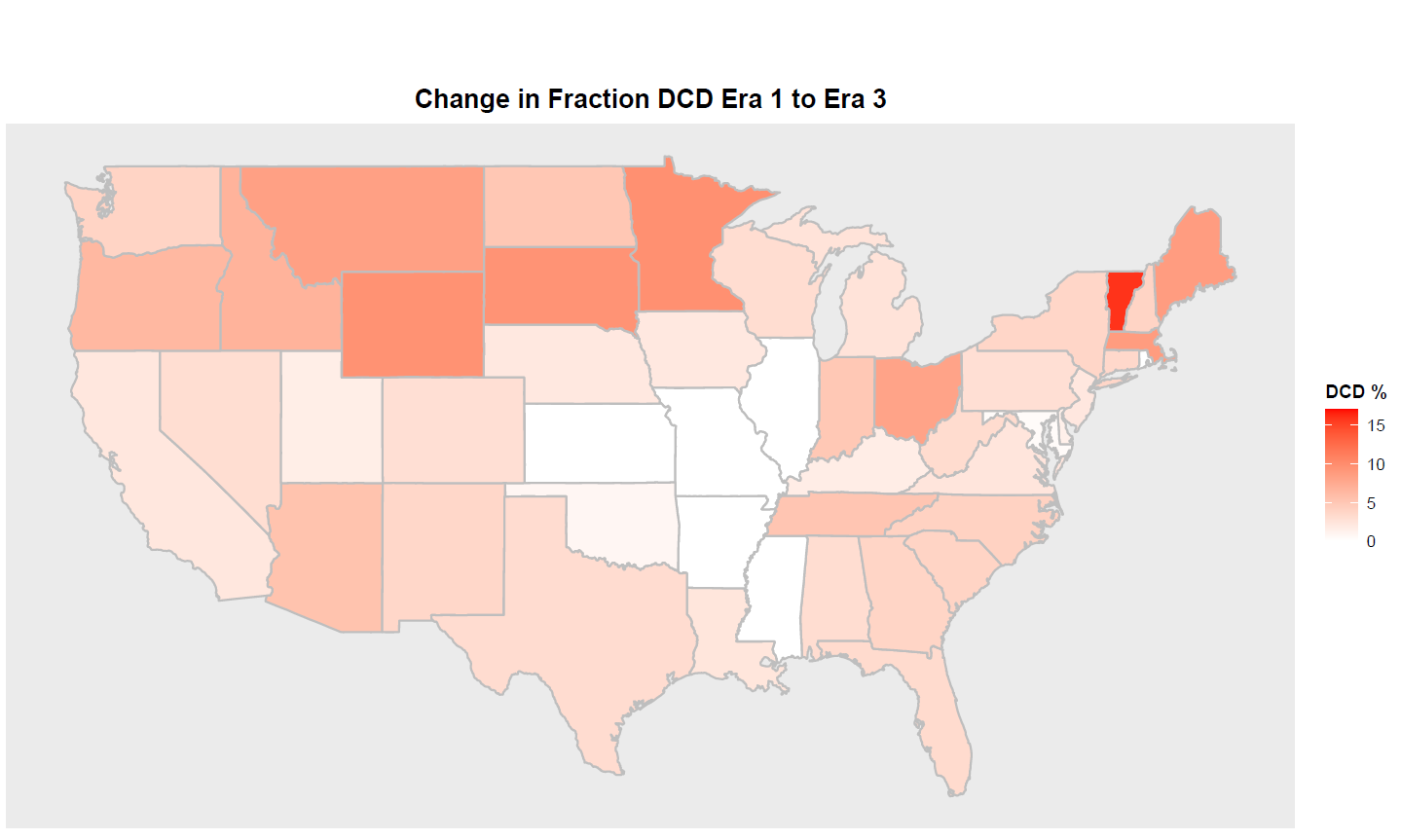

Supplement: Supplementary file 1 [file DataSheet1.DOCX]
